# Mitochondrial Transcription Factor A and Mitochondrial Genome as Molecular Targets for Cisplatin-Based Cancer Chemotherapy

**DOI:** 10.3390/ijms160819836

**Published:** 2015-08-20

**Authors:** Kimitoshi Kohno, Ke-Yong Wang, Mayu Takahashi, Tomoko Kurita, Yoichiro Yoshida, Masakazu Hirakawa, Yoshikazu Harada, Akihiro Kuma, Hiroto Izumi, Shinji Matsumoto

**Affiliations:** 1 Asahi Matsumoto Hospital, Kokuramimami-ku Tsuda, Kitakyushu-shi 800-0242, Japan; E-Mail: sm.kokura.1949@docomo.ne.jp; 2 Shared-Use Research Center, University of Occupational and Environmental Health School of Medicine, Yahatanishi-ku, Kitakyushu-shi 807-8555, Japan; E-Mail: kywang@med.uoeh-u.ac.jp; 3 Department of Neurosurgery, University of Occupational and Environmental Health School of Medicine, Yahatanishi-ku, Kitakyushu-shi 807-8555, Japan; E-Mail: maytak@med.uoeh-u.ac.jp; 4 Department of Gynecology, University of Occupational and Environmental Health School of Medicine, Yahatanishi-ku, Kitakyushu-shi 807-8555, Japan; E-Mail: t-kurita@med.uoeh-u.ac.jp; 5 Department of Gastroenterological Surgery, School of Medicine, Fukuoka University, Fukuoka 814-0180, Japan; E-Mail: yy4160@yahoo.co.jp; 6 Department of Radiology, Beppu Hospital, Kyushu University, Beppu 874-0838, Japan; E-Mail: hirakawa@beppu.kyushu-u.ac.jp; 7 Department of Pathology and Cell Biology, University of Occupational and Environmental Health School of Medicine, Yahatanishi-ku, Kitakyushu-shi 807-8555, Japan; E-Mail: y8harada@gmail.com; 8 Second Department of Internal Medicine, University of Occupational and Environmental Health School of Medicine, Yahatanishi-ku, Kitakyushu-shi 807-8555, Japan; E-Mail: akihiro_k@me.com; 9 Department of Occupational Pneumology, Institute of Industrial Ecological Science, University of Occupational and Environmental Health, Yahatanishi-ku, Kitakyushu-shi 807-8555, Japan; E-Mail: h-izumi@med.uoeh-u.ac.jp

**Keywords:** mtTFA, mtDNA, cisplatin, cancer chemotherapy, prognosis

## Abstract

Mitochondria are important cellular organelles that function as control centers of the energy supply for highly proliferative cancer cells and regulate apoptosis after cancer chemotherapy. Cisplatin is one of the most important chemotherapeutic agents and a key drug in therapeutic regimens for a broad range of solid tumors. Cisplatin may directly interact with mitochondria, which can induce apoptosis. The direct interactions between cisplatin and mitochondria may account for our understanding of the clinical activity of cisplatin and development of resistance. However, the basis for the roles of mitochondria under treatment with chemotherapy is poorly understood. In this review, we present novel aspects regarding the unique characteristics of the mitochondrial genome in relation to the use of platinum-based chemotherapy and describe our recent work demonstrating the importance of the mitochondrial transcription factor A (mtTFA) expression in cancer cells.

## 1. Introduction

Mitochondria produce the energy required to maintain cellular functions as well as command the process of apoptosis in order to inhibit these functions [1,2]. Cancer cells are immortalized as a result of various genetic and epigenetic changes in the genome. One of the major causes of cancer is the acquisition of the driving force of cellular proliferation induced by a mutation and/or the methylation of oncogenes and tumor suppressor genes. Another factor is resistance to apoptosis, which is also acquired due to genetic or epigenetic changes in apoptosis-related genes. These processes are closely related to the onset of drug resistance and the phenotypic characteristics of cancer stem cells. However, it is unknown whether the direct cytotoxic effects or apoptosis-inducing ability of anti-cancer agents are effective as cancer chemotherapy. Cancer cells are resistant to conventional chemotherapy and radiotherapy regimens, and various molecular mechanisms underlying the onset of resistance to therapy have been proposed [3,4]. For example, cellular survival signal transduction systems protect mitochondrial integrity against drug-induced stress, and apoptosis-related molecules are orchestrated around mitochondria to induce and execute apoptosis in cancer cells. These molecules also play important roles in the development of drug resistance in cancer cells. It is therefore intriguing to explore how the crosstalk of apoptosis-related molecules regulates various mitochondrial functions leading to drug resistance. This review describes some of the putative mechanisms of action of cisplatin at the site of the mitochondrial genome and in terms of the mtTFA expression, which may play an important role in the cellular functions of cancer cells and the prognosis of cancer patients.

## 2. Cisplatin and DNA

Cisplatin, cisplatinum or *cis*-diamminedichloroplatinum (II) is a well-known chemotherapeutic drug [5,6]. Among various anti-cancer agents, cisplatin is one of the most effective and widely used anti-cancer agents for the treatment of several types of solid tumors [7]. Special attention has been paid to its molecular mechanisms of action [8]. The cytotoxic effects of cisplatin are thought to be mediated primarily by the generation of nuclear DNA adducts, which, if not repaired, cause cell death as a consequence of the inhibition of DNA replication and transcription. However, the ability of cisplatin to induce nuclear DNA (nDNA) damage per se is not sufficient to explain its high degree of effectiveness.

## 3. Calcium Ion, Endoplasmic Reticulum (ER) Stress, ROS/Redox System and Apoptosis

Bcl-2 family proteins control mitochondrial outer membrane permeability and regulate apoptosis [9,10]. Among Bcl-2 family proteins, anti-apoptotic proteins, Bcl-2 and Bcl-xL localize at the mitochondria outer membrane and thereby inhibit cytochrome c release. On the other hand, apoptosis inducing pro-apoptotic proteins, Bad, Bid and Bax, localize from cytoplasm to mitochondria following various apoptosis signals and promote cytochrome c release [11]. Calcium ion is well-known to play an important role in the mitochondrial control of apoptosis [12].

It has been reported that cisplatin induces calpain-mediated Bid cleavage in association with increased intracellular calcium ion levels and calpain-cleaved Bid induces cytochrome c release from mitochondria [13]. The endoplasmic reticulum (ER) is a specialized organelle for the folding and trafficking of proteins and a cellular target of cisplatin [14]. ER stress signaling is involved in cisplatin-induced apoptosis, as cisplatin has been shown to induce the calpain-dependent activation of ER-specific caspase leading to apoptosis [15]. ER stress is also linked to the production of reactive oxygen species (ROS). ROS/Redox signaling is prominently associated with the progression of human malignancies [16]. Furthermore, ROS/Redox signaling is a critical stress response pathway associated with cancer chemotherapy. Oxidative damage is observed *in vivo* following exposure to cisplatin in several tissues, suggesting the role of oxidative stress in the pathogenesis of cisplatin-induced dose-limiting toxicities [17,18]. However, the mechanisms underlying the cisplatin-induced generation of ROS and their contribution to cisplatin cytotoxicity in normal and cancer cells remain poorly understood.

**Figure 1:**
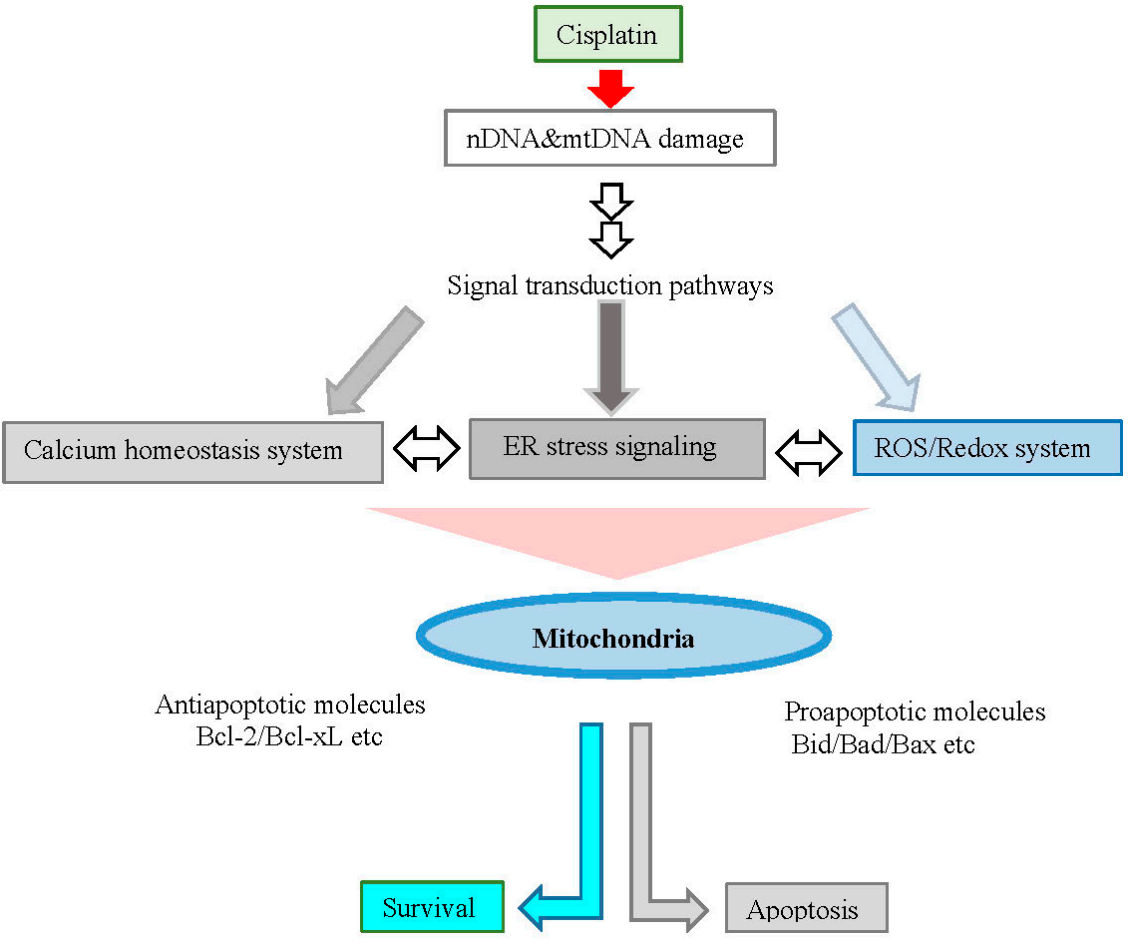
Cellular pathways and apoptosis induced by cisplatin. Cisplatin activates signal transduction pathways and may directly interact with mitochondria, which can induce apoptosis.

ROS generation occurs independently of the amount of cisplatin-induced nDNA damage and takes place in mitochondria as a consequence of the impairment of protein synthesis. The contribution of cisplatin-induced mitochondrial dysfunction in determining the cytotoxic effects of this drug varies among cells and depends on the mitochondrial redox status and integrity of mitochondrial DNA (mtDNA). Cisplatin-induced cellular pathways and responses are summarized in Figure 1.

## 4. mtTFA and Cisplatin Resistance

The development of resistance to cisplatin is a major obstacle in terms of the clinical outcomes of cancer patients [19,20,21]. Several mechanisms are thought to be involved in the onset of cisplatin resistance, including decreased intracellular drug accumulation, increased levels of cellular thiols, an increased nucleotide excision-repair activity and a decreased mismatch-repair activity [22,23]. Two transporters, copper-transporting P-type ATPase 7B (ATP7B) and ATP-binding cassette, sub-family C, member 2 (ABCC2), may be involved in cisplatin efflux and resistance [22,23]. Genome-wide analyses have been shown to be a powerful method for understanding drug resistance. A recent report showed that the Son of sevenless/the mitogen-activated protein kinase/extracellular signal-regulated kinase (SOS/MAPK/ERK) pathway is activated in cisplatin-resistant cells [24]. This pathway mediates the degradation of the proapoptotic molecule Bim resulting in the inhibition of mitochondria-dependent apoptotic pathways [25].

One antioxidative factor, the level of cellular glutathione, has been shown to be involved in the development of cisplatin resistance. Interestingly, upregulation of the ABC transporter and cellular glutathione is a characteristic of cancer stem cells (CSCs) [26,27,28]. Therefore, CSCs show multidrug resistance and radioresistance. Recently, it has been demonstrated that the expression of CD44, especially variant isoforms (CD44v) among major CSC markers, contributes to ROS defenses via upregulation of the synthesis of reduced glutathione [29]. These observations suggest that CSCs exhibit natural drug-resistant phenotypes and that drug-induced resistant cells display acquired resistant phenotypes. These data also indicate that CSCs mitochondria might differ from those of non-CSCs. However, little is known about the mitochondrial features related to energy production and the ROS/Redox system of CSCs. Therefore, defining these features will be critical for developing mitochondria-targeted therapeutics [30].

In general, the molecules responsible for these phenotypes are upregulated in cisplatin-resistant cells, which indicates that the transcription factors activated in response to cisplatin may play crucial roles in the development of drug resistance [19,20]. Interestingly, we have found that mitochondrial transcription factor A (mtTFA) is upregulated in cisplatin-resistant cells [31]. To date, several nuclear transcription factors involved in the development of resistance against platinum-containing agents have been identified, including Y-box binding protein-1 (YB-1) [32], nuclear factor I/B [33], activating transcription factor 4 (ATF4) [34], zinc-finger factor 143 (ZNF143) [35], and Clock [36]. Several of these transcription factors are regulated by E-box binding transcription factors, which regulate the epithelial-mesenchymal transition [37]. Furthermore, both YB-1 and ZNF143 lack the high-mobility group (HMG) domain and are capable of binding preferentially to cisplatin-modified DNA in addition to HMG domain proteins, such as mtTFA. It has therefore been proposed that various mitochondrial functions may be regulated by the circadian clock system [38], which controls cancer cell proliferation and angio/stromagenesis via WNT signaling [39,40].

Mitochondrial dysfunction is associated not only with cancer progression, but also with chemoresistance [41], and a relationship of mtDNA mutations with cisplatin-induced apoptosis and/or cisplatin resistance has been reported. These mtDNA mutations endow cancer cells with chemoresistance [42]. It has also recently been reported that nuclear co-activators, including peroxisome proliferator-activated receptor gamma co-activator-1 (PGC-1), are upregulated and compensate for respiratory chain defects due to mtDNA mutations in cisplatin-resistant cells [43]. Interestingly, a frameshift mutation of the nuclear mtTFA gene has been reported in colorectal cancer cells with microsatellite instability [44]. These cancer cells express truncated mtTFA and show more resistance to cisplatin-induced apoptosis. These data also show that mitochondrial dysfunction due to genetic changes in both mtDNA and nDNA is closely associated with chemoresistance.

## 5. Mitochondrial DNA as a Target for Cisplatin

Mitochondria are closely related to carcinogenesis, cancer progression and chemoresistance. The genes encoded by mtDNA are tightly packed together with minimal noncoding regions. Although the number of mitochondria and mtDNA in cancer cells has not been extensively studied, a reduction in the mtDNA copy number has been reported in various human cancers [45]. Furthermore, it is well known that mtDNA is more susceptible than nDNA to damage from reactive oxygen species and chemicals, including anti-cancer agents, due to either a limited capacity for DNA repair or the presence of nucleosome-free structures [46,47].

Cisplatin is a major DNA-targeting agent and the most potent key drug for treating solid tumors among anti-cancer agents. It has been shown that the mitochondrial DNA adduct levels are higher than the nDNA adduct levels and that both a higher degree of initial binding and lack of removal of cisplatin-DNA adducts appear to contribute to the preferential formation of cisplatin-mtDNA adducts. The effects of cisplatin arise from its ability to damage DNA, with the major adducts formed being intrastrand d(GpG) crosslinks. Therefore, the target DNA sequence of cisplatin is G-stretch. Table 1 shows the number of G-stretch sequences in the mtDNA of various species in comparison with that observed in nDNA. As shown in Table 1, the number of G-stretch sequences is higher in primates than in other species. Furthermore, the number of G-stretch sequences in mtDNA is significantly higher than that noted in nDNA. These findings indicate that the number of G-stretch sequences may be related to the higher initial binding capacity of cisplatin to mtDNA.

**Table 1:** Number of cisplatin-targeted DNA sequences in mitochondrial DNA.

Species	Total Number of mtDNA		GG	GGG	GGGG	GGGGG
Human	16,565	L chain	426	73	15	4
		H chain	1772	624	224	69
		total	2198	697	239	73
Gorilla	16,364	L chain	425	71	16	5
		H chain	1712	596	216	72
		total	2137	667	232	77
Rat	16,300	L chain	397	63	14	4
		H chain	1299	377	99	32
		total	1696	440	113	36
Mouse	16,300	L chain	397	58	11	3
		H chain	1104	288	72	19
		total	1501	346	83	22
Xenopus	17,553	L chain	445	72	12	1
		H chain	1091	259	60	8
		total	1536	331	72	9
Drosophila	16,019	L chain	599	230	16	1
		H chain	770	358	78	30
		total	1369	588	94	31
Expectation		Double strand	2071	518	129	32
		Single strand	1035	259	65	16
Nuclear DNA		Double strand	1919 ± 319	501 ± 77	118 ± 25	35 ± 14

Expectation indicates the calculated number of each G-stretch sequence in the same number of nucleotide sequences of human mitochondria. Nuclear DNA indicates the average number of G-stretch sequences in human *YB-1*, *Sp-1* and *ZNF143* genes by way of example.

It has been demonstrated that the formation of DNA adducts is increased under acidic conditions [48]. In general, perturbation of the intracellular pH of highly proliferative cancer cells is observed [49]. If the pH around mtDNA is higher than that noted around nDNA, this finding may explain the higher initial binding capacity of cisplatin to mtDNA.

## 6. Cellular Functions of mtTFA

mtTFA is a 25-kDa protein encoded by a nuclear gene and imported to the mitochondria, where it is required for both the transcription and maintenance of mitochondrial DNA. mtTFA preferentially recognizes cisplatin-damaged DNA as well as oxidized DNA. Increased apoptosis is observed in mtTFA knockout animals, suggesting that mtTFA is involved in the process of apoptosis. However, the roles of mtTFA have not been extensively studied in cancer cells [50]. We recently reported the nuclear localization of mtTFA [51]. In addition, the proportion of nuclear-localized mtTFA varies among different cancer cells, and DNA microarray and chromatin immunoprecipitation assays have shown that mtTFA regulates the transcription of nuclear genes. Furthermore, the overexpression of mtTFA enhances the growth of cancer cell lines, whereas the downregulation of mtTFA inhibits the growth of these cells by regulating mtTFA target genes, such as baculoviral IAP repeat-containing 5 (BIRC5; also known as survivin) [52] and BCL2L [53]. Moreover, knockdown of the mtTFA expression induces p21-dependent G1 cell cycle arrest. These results imply that mtTFA functions in both nuclei and mitochondria to promote cell growth.

mtTFA is upregulated by treatment with cisplatin as well as the tumor suppressor p53. p53 is a multifunctional tumor suppressor protein that interacts with a variety of proteins with both positive and negative effects. Furthermore, mutation of the p53 gene is often observed in a wide variety of tumors. Mutated p53 gene products are stabilized and accumulate in the cytoplasm of cancer cells. A fraction of p53 proteins localize in the mitochondria at the onset of p53-dependent apoptosis, although not during p53-independent apoptosis [54]. Using immunochemical coprecipitation, we observed the binding of mtTFA with p53 [55]. The interaction between mtTFA and p53 requires the high mobility group-box B1 or high mobility group-box B2 of mtTFA and amino acids 363–376 of p53. In addition, the binding of mtTFA to cisplatin-modified DNA is significantly enhanced by p53, whereas binding to oxidized DNA is inhibited. Meanwhile, mtTFA preferentially recognizes cisplatin-damaged DNA, as well as oxidized DNA, and the conformational alteration induced by 8-oxo-dG in DNA differs from that induced by cisplatin. Therefore, the different effects of p53 on the binding of mtTFA may depend on structural differences in damaged DNA. Further analyses of the mechanisms by which mtTFA recognizes damaged DNA would be desirable for understanding the differential modulation of mtTFA binding by p53.

The interaction between mtTFA and p53 may be involved in transcriptional regulation, and, similar to transcription factors, both proteins have a DNA-binding domain. Our findings suggest that the interaction of p53 with mtTFA may play an important role in apoptosis. In addition, apoptosis is increased in cells lacking mtTFA [56]. These results suggest a model in which p53 inhibits the ability of mtTFA to execute cell death signaling. Hence, it is possible that manipulating the mtTFA function may be used as a mitochondria-targeted cancer treatment to enhance apoptosis.

It has previously been demonstrated that the expression levels of both mtTFA and mitochondrial antioxidant protein thioredoxin2 (TRX2) are upregulated in cisplatin-resistant cell lines [31]. In addition, TRX2 directly interacts with mtTFA and enhances its damaged DNA binding activity. These results suggest that TRX2 functions as an antioxidant as well as supporting the mtTFA function.

**Figure 2:**
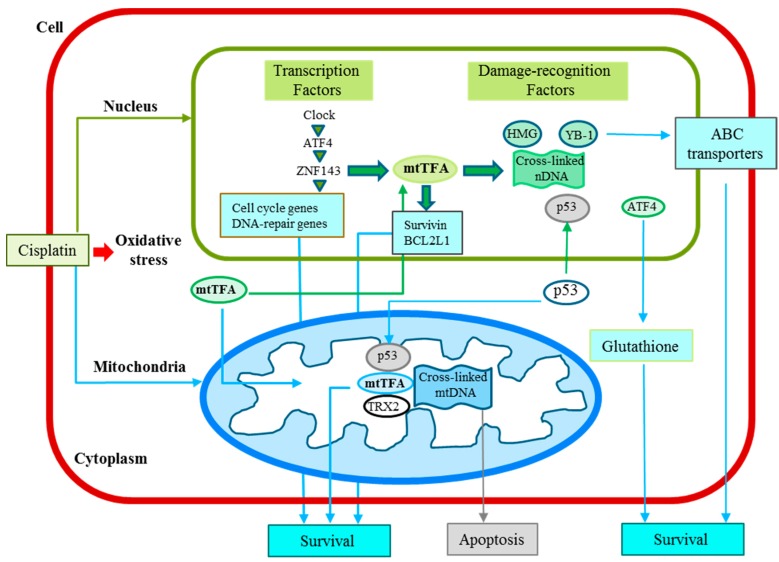
Cisplatin-induced signaling, cisplatin resistance and transcription system in cancer cells discussed in this review. mtTFA functions in both nuclei and mitochondria to not only interact with cisplatin-modified DNA, but also regulate the nuclear and mitochondrial gene expression.

ZNF143 has been shown to be a cisplatin-inducible gene that regulates the mitochondrial ribosomal protein S11 [57]. Interestingly, there is one ZNF143 binding region in the promoter region of the mtTFA gene. The involvement of ZNF143 in cell growth and the protection of cells from oxidative damage, as well as the effects of cisplatin treatment, has recently been reported [35,58,59]. Furthermore, a strong ZNF143 expression was previously found to show a significant correlation with pathologically moderate to poor differentiation and highly invasive characteristics in 183 paraffin-embedded tumor samples from patients with lung adenocarcinoma [60]. Based on these reports, we next discuss the clinical implications of mtTFA. A schematic summary of the mutual relationship of transcription factors, including mtTFA, is shown in Figure 2 and the major factors described in this review are listed with detailed information in Table 2.

**Table 2:** Selected transcription factors associated with drug resistance and target genes. “+” means that factors can recognize cisplatin-crosslinks or can render cells resistant.

Factors	Selected Target Genes	Selected Interacting Factors	Damage Recognition	Drug Resistance	Other Functions	References
mtTFA	*Survivin BcLL2*	p53, TRX2	+	+	Cell growth, anti-apoptosis	[54,55]
ZNF143	*mtTFA*	p73	+	+	Cell cycle, DNA repair	[35,57]
ATF4	*ZNF143*			+	Glutathione biosynthesis	[34,36]
Clock	*ATF4*, *Tip60*			+	Circadian rhythm, DNA repair	[36,38]
YB-1	*MDR1*	p53, PCNA, Topo1	+	+	Endothelial cell growth	[61,62,63]

## 7. Clinical Implications of the mtTFA Expression in Tumors

Mitochondrial transcription factor A (mtTFA) is necessary for both the transcription and maintenance of mitochondrial DNA (mtDNA). However, mtTFA is also localized in the nucleus and regulates nuclear genes. To date, the expression of mtTFA has not been thoroughly elucidated in the clinical setting. Studies of clinical specimens have recently investigated the relationships between clinicopathological factors, the prognosis and the immunohistochemical expression of mtTFA, as shown in Table 3.

**Table 3:** Associations of the mTFA expression and malignant characteristics in human cancers. B cell lymphoma 2 like 1 (BCL2L1), Folinic acid plus 5-fluorouracil plus oxaliplatin (FOLFOX).

Tumor	No. of Cases	Malignant Characteristics	Reference
Serous ovarian	60	Poor prognosis, up-regulation of BCL2L1 expression	[64]
Pancreatic ductal adenocarcinoma	70	Poor prognosis, up-regulation of survivin expression	[65]
Colorectal	105	Poor prognosis	[66]
Metastatic colorectal	59	Poor clinical outcome with FOLFOX treatment	[67]
Endometrial	245	Invasion and metastasis, p53 mutation, poor prognosis	[68]

An immunohistochemical analysis of the mtTFA expression in 60 tissue samples of serous ovarian cancer showed 56.7% of the serous ovarian cancer patients to be positive for mtTFA, whereas 43.3% were negative [64]. A significant correlation was also reported between the nuclear mtTFA expression and the BCL2L1 expression in seven ovarian cancer cell lines as well as specimens of clinical ovarian cancer. Furthermore, a univariate survival analysis showed that the overall five-year survival rate is significantly worse for patients with mtTFA-positive cancer *versus* mtTFA-negative cancer.

The correlations between the mtTFA expression and the survivin index as well as a poor prognosis were recently assessed using 70 paraffin-embedded tumor samples from patients with surgically-resected pancreatic adenocarcinoma [65]. The results suggested that mtTFA is a prognostic factor for a poor outcome of human cancer and may function as an antiapoptotic factor, regulating target genes, such as BCL2L1 and survivin. In another study, clinical specimens from 105 colorectal patients were immunohistochemically stained using a polyclonal anti-mtTFA antibody [66]. Consequently, a total of 47 (44.8%) of the 105 patients with colorectal cancer were determined to have a positive mtTFA expression, and a positive expression of mtTFA was found to significantly correlate with both lymph node and distant metastasis in addition to an advanced TNM stage. Furthermore, the survival of the patients with a positive mtTFA expression was significantly worse than that of the patients with a negative mtTFA expression. Therefore, a positive mtTFA expression appears to be a useful marker of tumor progression and a poor prognosis in patients with colorectal cancer.

Whether the expression of mtTFA predicts the clinical outcomes of patients with metastatic colorectal cancer treated with modified 5-fluorouracil, leucovorin and oxaliplatin 6 (mFOLFOX6) was recently evaluated [67]. In that study, 59 patients with metastatic lesions of colorectal cancer treated with mFOLFOX6 were analyzed. As a result, a strong expression of mtTFA was detected in eight of 33 cases of a complete response/partial response (24.2%) and 18 of 26 cases of SD/PD (69.2%), indicating that the mtTFA expression significantly correlates with the response to chemotherapy (*p* < 0.01). These results suggest that immunohistochemical studies of mtTFA may be useful for predicting the clinical outcomes of metastatic colorectal cancer patients treated with FOLFOX.

The relationships between the immunohistochemical expression of mtTFA and various clinicopathological variables in 245 cases of endometrioid adenocarcinoma were also recently evaluated [68]. In that report, the mtTFA expression in the endometrioid adenocarcinomas was shown to be significantly associated with the surgical stage, myometrial invasion, lymphovascular space invasion, cervical invasion and lymph node metastasis. In addition, a correlation analysis between the mtTFA and p53 expression levels using the Pearson test showed a significant correlation, and a univariate survival analysis showed that the 10-year overall survival rate of the patients with mtTFA-positive endometrioid adenocarcinoma was significantly worse than that of the patients with mtTFA-negative endometrioid adenocarcinoma. Therefore, a positive mtTFA expression may be a useful marker of tumor progression and a poor prognosis in patients with endometrioid adenocarcinoma.

## 8. Conclusions and Perspectives

Recent studies have demonstrated the important role of mitochondria in cancer biology. This review focused on the mitochondrial genome and transcription factor A. Since mtTFA proteins have been shown to be highly expressed in cancer and drug-resistant cells compared to normal cells, and the mtTFA expression is upregulated by signals of oxidative and DNA damage stress, this protein may potentially serve as a promising target in cancer chemotherapy. Promising strategies include inhibition of the expression and/or function of mtTFA specifically in cancer cells. Small interference RNA can inhibit specific gene expression levels. mtTFA functions with other transcription factors such as mtTFB1 and B2. Inhibiting the interaction with mtTFBs is expected to be a good strategy using peptides, and both RNA and peptide drugs are good candidates using cancer-specific drug delivery systems [69,70]. Furthermore, the mtTFA expression and mitochondrial genome possess attractive characteristics for platinum-based chemotherapy, and the mtTFA expression reflects a poor prognosis in patients with solid cancers. These findings indicate that further research on mitochondria may provide novel and unique therapeutic interventions for overcoming cancer.
